# Comparison of high-grade human papillomavirus-induced cervical lesions between human immunodeficiency virus-positive and human immunodeficiency virus-negative women

**DOI:** 10.1590/1806-9282.20250214

**Published:** 2025-09-19

**Authors:** Flávia Werner da Rocha Jesuino, Edward Araujo, Levon Badiglian-Filho

**Affiliations:** 1 A.C. Camargo Câncer Center, Fundação Antônio Prudente, Department of Gynecologic Oncology – São Paulo (SP), Brazil.; 2 Universidade Federal de São Paulo, Escola Paulista de Medicina da, Department of Obstetrics – São Paulo (SP), Brazil.; 3 Universidade Municipal de São Caetano do Sul, Discipline of Woman Health – São Caetano do Sul (SP), Brazil.

**Keywords:** Sexually transmitted diseases, Human papillomavirus, Cervical neoplasms, Human immunodeficiency virus

## Abstract

**OBJECTIVE:**

The aim of this study was to compare the evolution of high-grade human papillomavirus-induced cervical lesions between human immunodeficiency virus-positive and human immunodeficiency virus-negative women.

**METHODS:**

Human immunodeficiency virus-positive and human immunodeficiency virus-negative women were enrolled in a retrospective cohort study after undergoing large-loop excision of the transformation zone between January 2008 and December 2017 as participants in the high-grade cervical lesion screening program. The evolution of human papillomavirus lesions induced in human immunodeficiency virus-infected women was evaluated in comparison with the general population. The following potentially confounding variables were examined: age at treatment and at the end of follow-up, histologic grade of treated intraepithelial disease, compromised margins, adequacy of colposcopy during follow-up, and recurrence of histopathologic diagnosis of high-grade squamous intraepithelial lesion.

**RESULTS:**

Of the 195 women, 51 were human immunodeficiency virus-positive (26.2%) and 144 were human immunodeficiency virus-negative (73.8%). Age of menarche (11.8±1.5) and sexarche (14.5±2.2) were earlier in human immunodeficiency virus-positive women (p=0.021 and 0.006, respectively). Parity (2.35±109) and number of pregnancies (2.86±1.47) were higher in human immunodeficiency virus-positive women (p=0.027 and 0.018, respectively). The prevalence of incomplete schooling was lower in human immunodeficiency virus-positive women (47.1 vs. 19.8%, p=0.002). Smoking was more common in human immunodeficiency virus-positive women (58.8 vs. 31.9%, p=0.001). Compromised surgical margins were found in 33.3% of human immunodeficiency virus-positive and 16.7% of human immunodeficiency virus-negative women (p<0.001). The risk of recurrence was 37.3% in human immunodeficiency virus-positive and 13.9% in human immunodeficiency virus-negative women (p<0.001). Cumulative survival over months was shortened in human immunodeficiency virus-positive women (p<0.001).

**CONCLUSION:**

human immunodeficiency virus-positive women in this cohort had a significantly higher risk of recurrence than human immunodeficiency virus-negative women in addition to shorter overall survival.

## INTRODUCTION

Cervical cancer is the fourth most common cancer in women, with most cases occurring in less developed regions^1^. Infection with oncogenic human papillomavirus (HPV) subtypes is the primary causal factor for the development of invasive cervical neoplasia and is identified in almost all cervical cancers^2,3^. This cancer is almost exclusively (99.7%) associated with persistent infection by HPV types with high oncogenic potential, mainly types 16 and 18^4,5^. HPV is acquired through sexual transmission, often at a young age, and is one of the most common sexually transmitted diseases in adolescents, with a prevalence of 30–50% in this age group^6^.

The natural history of cervical intraepithelial neoplasia shows that it is a multifactorial process in which HPV is necessary but not sufficient to cause the appearance of the lesion^7^. HPV E6 and E7 oncoproteins are known to be the second most important oncogenic proteins for cervical carcinogenesis and are known to activate oncogenic pathways and repress tumor suppressor pathways^8^. Degradation of p53 by increasing telomerase expression through E6 and binding to retinoblastoma protein through E7 results in host cell cycle dysregulation and cell growth^5^. Other important cofactors include immunosuppression (especially human immunodeficiency virus [HIV]), smoking, parity (higher number of full-term pregnancies increases risk), and oral contraceptive use^9^. Girls and young women aged 15–24 years are twice as likely to be living with HIV as young men, and about 4,900 women aged 15–24 years become infected with HIV each week^10^.

Compared with HIV-negative women, HIV-positive women are more likely to be infected with HPV, to have persistent HPV that leads to precancer, to have more difficult-to-treat precancerous lesions, to have larger lesions, to have higher rates of treatment recurrence, and to have precancerous lesions that progress more rapidly to invasive cancer^1,11^.

Hence, the objective of this study was to compare the evolution of high-grade HPV-induced cervical lesions between HIV-positive and HIV-negative women.

## METHODS

A retrospective comparative cohort study was conducted on women with and without HIV infection who underwent large-loop excision of the transformation zone (LLEZT) for the treatment of high-grade squamous intraepithelial lesion (HSIL), between January 2008 and December 2017. Women were enrolled from the time of treatment and followed up with colpocytology and colposcopy. This was a multicenter study with the participation of HIV-positive and HIV-negative women from Itajaí, SC (Center for Infectious Diseases) and São Paulo, SP (AC Camargo Cancer Center). The study was approved by the Ethics Committee of AC Camargo Cancer Center (n° 2725/19).

The study group consisted of HIV-positive women diagnosed with HSIL who were treated with LLETZ, with no history of previous treatment, and who underwent at least one cytologic and colposcopic evaluation 6 months after treatment. The control group consisted of HIV-negative women diagnosed with HSIL.

The information was collected on the study factor and outcome, and on confounding variables associated with the study factor and/or outcome that may have influenced this relationship. Thus, the following variables were measured: presence of HIV; diagnosis of recurrent HSIL confirmed histologically at any time during follow-up after LLETZ; age at treatment and at end of follow-up; histologic grade of intraepithelial disease treated by LLETZ (before enrollment); surgical margin involvement in the segment excised by LLETZ (possible incomplete excision) as reported by histopathologic examination (before enrollment); CD4 T-lymphocyte count; and adherence to antiretroviral therapy. All these variables were obtained at the time of diagnosis/treatment and at each follow-up visit after treatment. Pap smears were obtained on these occasions, and the results of these tests were used as a guide at the next appointment. Biopsy was performed in cases of large colposcopic changes or small changes with cytologic findings suggestive of HSIL. If the squamocolumnar junction was not completely visible and the cytology showed high-grade atypia, cervical conization was indicated. Recurrence was considered to have occurred if HSIL was diagnosed in histologic specimens obtained by colposcopically guided biopsy or other surgical procedures (LLETZ or conization). Information on HIV-related disease was obtained from electronic medical records or by reviewing the medical records in the services where patients were monitored and treated for their HIV infection. The interventions considered were those closest to the follow-up visits. At each visit, data related to cervical disease and HIV infection, if applicable, were collected.

The data were stored in RedCap and then processed using the statistical packages Epi-Info version 6.4d and Stata version 10.0. Descriptive statistics were used to characterize the study population. Inferential statistics were used to compare variables between women with and without HIV. Categorical variables were compared using the chi-squared test or Fisher's exact test, depending on the expected values obtained from the contingency tables. For numerical variables, Student's t-test and Mann-Whitney test were used to compare means for unpaired groups. To estimate the incidence and relative risk of recurrence of preinvasive lesions, we used a measure of cumulative incidence in the first and second years and incidence density over the course of the study in each group. To estimate the risk of recurrence over time, we used the Kaplan-Meier curve. The ignificance level for all statistical tests was 5%.

## RESULTS

A total of 195 women met the inclusion criteria, of whom 51 were HIV-positive (26.2%) and 144 were HIV-negative (73.8%). Table 1 shows age and variables related to reproductive and sexual history. Significant statistical differences were observed between the groups with respect to age, menarche, sexarche, and parity.

**Table 1:** Age and variables related to reproductive and sexual history.

Variables		HIV-negative	HIV-positive	p-value
Age (years)				
	n	144	51	0.78
	Mean	37.21	35.46	
	Standard deviation	11.75	8.32	
	Minimum–maximum	19.02–78.57	19.01–51.52	
Menarche age (years)				
	n	112	20	0.021
	Mean	12.87	11.85	
	Standard deviation	1.67	1.56	
	Minimum–maximum	8–17	9–15	
Sexarche age (years)				
	n	98	20	0.006
	Mean	16.58	14.6	
	Standard deviation	3.27	2.23	
	Minimum–maximum	12–36	9–19	
Menopausal age (years)				
	n	22	3	0.23
	Mean	48.14	46.64	
	Standard deviation	5.05	2.08	
	Minimum–maximum	32–55	45–49	
Parity				
	n	136	51	0.027
	Mean	2.10	2.35	
	Standard deviation	2.3	1.09	
	Minimum–maximum	0–20	0–6	
Abortion				
	n	136	51	0.34
	Mean	0.33	0.49	
	Standard deviation	0.70	0.90	
	Minimum–maximum	0–5	0–4	

HIV: human immunodeficiency virus.

The prevalence of incomplete schooling was lower in HIV-positive women (47.1 vs. 19.8%, p=0.002). There were no significant differences between HIV-positive and HIV-negative groups regarding chronic hypertension (11.8 vs. 13.9%, p=0.88). Smoking was more common in HIV-positive women (58.8 vs. 31.9%, p=0.001).

The mean CD4 T-lymphocyte count was 337.15±192.72 cells/mm^3^ (range, 85–940). Considering the following cutoff points for CD4+ count (cells/mm^3^) in the HIV-positive group, <200, 200–400, and ≥400, the relapse/persistence rates were 38.5% (10), 30.8% (8), and 30.8% (8), respectively. Regarding adherence to antiretroviral treatment among the 51 HIV-positive women, the majority (76.5%) reported irregular treatment adherence at the time of HSIL diagnosis, while 23.53% reported regular treatment visits.

In relation to the results of the diagnostic Pap smears, the following were found in HIV-negative and HIV-positive patients, respectively: low-grade squamous intraepithelial lesion (LSIL) in 11.1 and 21.6%; HSIL in 75 and 64.7%; atypical squamous cells of undetermined significance (ASCUS) in 6.9 and 13.7%; and atypical glandular cells of undetermined significance (AGUS) represented 1.4 and 0%.

The anatomopathologic result of the surgical specimen of the lesion was of high grade in 71.5% of HIV-negative and 72.5% of HIV-positive women. The surgical specimen was negative in 11.8% of HIV-negative and 1% of HIV-positive women, and it was LSIL in 9 and 9.8% of HIV-negative and HIV-positive women, respectively. Invasive squamous cell carcinoma was found in 6.9% of HIV-negative and 15.7% of HIV-positive women. The endocervical surgical margin was compromised in 11.8% of pathology specimens from HIV-negative women and in 37.3% of specimens from HIV-positive women (p<0.001).

The most common treatment was the LLETZ alone in 82.6 and 90.2% of HIV-negative and HIV-positive cases, respectively. Classic cone was performed in 13.9 and 9.8% of HIV-negative and HIV-positive cases, respectively. None of the HIV-positive patients received LLETZ associated with laser, conization associated with laser, or hysterectomy.

With regard to recurrence during the follow-up period after excision of the HSIL, 13.9% of HIV-negative and 37.3% of HIV-positive women had recurrence. In addition, 6.9 and 19.6% of HIV-negative and HIV-positive women, respectively, had persistence of the lesion, considered if the lesion was detected less than 1 year after the initial lesion was excised. Using the logistic regression results, the odds ratio (OR) for lesion recurrence was 3.68 times higher in HIV-positive women (95%CI 1.75–7.70). For lesion persistence in HIV-positive women, the OR was 3.26 (95%CI 1.27–8.39).

Of the 59 recurrences or persistence of the lesion, 14 were not treated, all of them in the HIV-positive category, which represented 23.7% of the women. Table 2 shows the type of treatment in cases of lesion recurrence, without any statistical difference between HIV-positive and HIV-negative groups.

**Table 2:** Type of treatment in cases of lesion recurrence.

Recurrence treatment		HIV, n (%)		Total (n)/valid percentage (%)	p-value
		HIV-negative	HIV-positive		
Cone					
	No	143 (99.3)	51 (100)	194 (99.5)	1.0
	Yes	1 (0.7)	0 (0)	1 (0.5)	
	Total	144 (100)	51 (100)	195 (100)	
LLEZT					
	No	141 (97.9)	51 (100)	192 (98.5)	0.56
	Yes	3 (2.1)	0 (0)	1 (1.5)	
	Total	144 (100)	51 (100)	195 (100)	
Hysterectomy					
	No	128 (88.9)	46 (90.2)	174 (89.2)	1.0
	Yes	16 (11.1)	5 (9.8)	21 (10.8)	
	Total	144 (100)	51 (100)	195 (100)	
Follow-up					
	No	137 (95.1)	50 (98)	187 (95.9)	0.68
	Yes	7 (4.9)	1 (2)	8 (4.1)	
	Total	144 (100)	51 (100)	195 (100)	
Radiotherapy					
	No	143 (99.3)	48 (94.1)	191 (97.9)	0.056
	Yes	1 (0.7)	3 (5.9)	4 (2.1)	
	Total	144 (100)	48 (100)	191 (97.9)	
Chemotherapy					
	No	143 (99.3)	48 (94.1)	191 (97.9)	0.056
	Yes	1 (0.7)	3 (5.9)	4 (2.1)	
	Total	144 (100)	51 (100)	195 (100)	
Brachytherapy					
	No	144 (100)	49 (96.1)	193 (99.0)	0.067
	Yes	0 (0)	2 (3.9)	2 (1.0)	
	Total	144 (100)	51 (100)	195 (100)	

LLEZT: large-loop excision of the transformation zone; HIV: human immunodeficiency virus.

**Figure 1:**
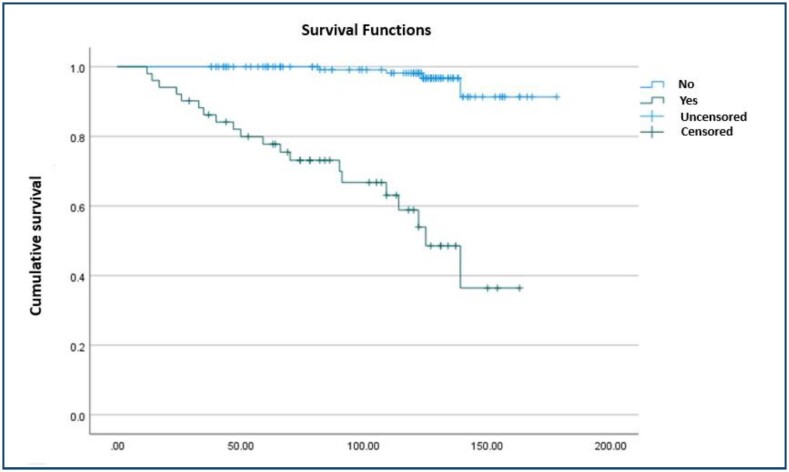
Cumulative survival over the months of follow-up between human immunodeficiency virus-negative and human immunodeficiency virus-positive women (p<0.001).

After follow-up, which ranged from 3 to 10 years, 0.7% of HIV-negative and 23.5% of HIV-positive women died of cervical cancer; 2.1% of HIV-negative and none of HIV-positive women died of other causes; 15.7% died of HIV; and 87.6% remained alive. Survival over the months of follow-up is shown in Figure 1, with a decline in survival after the fourth year of control, with a significant difference for the HIV-positive women (p<0.001).

## DISCUSSION

This study found that the majority of women in both groups started having sex in adolescence, but HIV-positive women had a lower mean age of sexual initiation compared to HIV-negative women. Kjellberg et al.^12^ found a significant increase in the risk of HPV infection associated with sexual initiation before the age of 15.

In terms of risk factors, Kunckler et al.^13^ found that women with a history of six or more sexual partners were 2.12 times more likely to be infected with HPV. The number of children was a facilitating factor for HPV infection, and women with more than two children were 0.66 times more likely to have HPV infection than women with one or no children. This study also found differences between the groups in the number of pregnancies and parity, which were significantly higher in the HIV-positive group.

Another variable examined was schooling, which is an important baseline indicator of health and can be used as a socioeconomic marker. Gaspar et al.^14^ found a statistically significant association between low formal education and HIV seropositivity, indicating 4.384 times greater likelihood of HIV seropositivity for women who studied between 0 and 4 years and 2.530 times greater likelihood of HIV seropositivity for women who studied between 5 and 8 years. In our study, 47.1% of the HIV-positive women reported that they had no formal education or had not completed primary school.

Leo Nogueira Costa et al.^15^ observed an increased risk of cancer in the HIV-positive population, associated with the fact that this population has higher rates of smoking. In addition, the oncogenic potential of tobacco use may be prolonged in HIV-positive individuals for up to 5 years after smoking cessation. In our study, there was a statistical difference in smoking habits between HIV-negative and HIV-positive women, with the habit being more prevalent in HIV-positive women. Smoking has been identified as an important cofactor in the development of cervical neoplasia. Kjellberg et al.^12^, in a study of HIV women with a sample of approximately 400 participants, identified smoking as the most important environmental risk factor for cervical neoplasia.

HPV infection is most often transient, with only 10% of people developing persistent infection, that is, for more than a year, and when it persists, it is considered the most common risk factor for cervical cancer^16^. Debeaudrap et al.^17^ also described the risk of recurrence or persistence after treatment with loop electrosurgical excision procedure (LEEP) at 10%, and this risk increased in patients with positive margins. In our study, the recurrence rate was 13.9 and 37.3% in HIV-negative and HIV-positive women, respectively. The study by Russomano et al.^18^ found a 42% higher risk of HSIL recurrence in HIV-positive women compared to HIV-negative women. These findings are also consistent with the study by Gilles et al.^19^, in which the persistence of cervical disease was more frequently observed in HIV-positive women (42%) and cone biopsy margins were more frequently compromised in HIV-positive women (37%) than in HIV-negative women. Furthermore, recurrence may be associated with treatment adherence in HIV-positive women, as in our study, regular treatment adherence was reported by only 23.53% of HIV-positive women. Carlander et al.^20^ performed a cohort study on 179 HIV-positive women and women with adenocarcinoma in situ or cervical cancer, with follow-up between 1983 and 2015. In their study, treatment failure was defined as the presence of CIN2+ at the initial follow-up. Suppressive antiretroviral therapy at the time of treatment of CIN2+ was associated with a reduced OR for treatment failure. Immunosuppression (CD4 cell count <200 cells/mm^3^) was strongly associated with treatment failure.

In this study, there was no difference in the type of treatment administered between the groups. Debeaudrap et al.^17^ evaluated 40 studies on the prevalence of treatment failure in HIV-infected women and found no significant difference in the prevalence of treatment failure between cryotherapy and electrosurgical excision for LEEP. However, there was a significant increase in treatment failure in HIV-positive compared with HIV-negative women.

Studies from the United States and Botswana suggest that survival may be worse for HIV-infected people compared to HIV-uninfected women with cervical cancer^21^. In this study, there was a significant difference in survival between HIV-negative and HIV-positive women; at the end of follow-up, 60.8% of HIV-positive women were alive compared to 97.2% of HIV-negative women.

## CONCLUSION

HIV-positive women with high-grade HPV-related cervical lesions had higher rates of lesion recurrence and surgical margin involvement, as well as lower overall survival, compared to HIV-negative women.

## Data Availability

The datasets generated and/or analyzed during the current study are available from the corresponding author upon reasonable request.
